# Virtual reality check: a comparison of virtual reality, screen-based, and real world settings as research methods for HRI

**DOI:** 10.3389/frobt.2023.1156715

**Published:** 2023-06-27

**Authors:** Jana Plomin, Paul Schweidler, Astrid Oehme

**Affiliations:** ^1^ Fraunhofer Fokus, Digital Public Services, Berlin, Germany; ^2^ HFC Human-Factors-Consult GmbH, Berlin, Germany

**Keywords:** virtual reality, human-robot interaction, method, embodiment, service robot, human-centered design (HCD)

## Abstract

To reduce costs and effort, experiments in human-robot interaction can be carried out in Virtual Reality (VR) or in screen-based (SB) formats. However, it is not well examined whether robots are perceived and experienced in the same way in VR and SB as they are in the physical world. This study addresses this topic in a between-subjects experiment, measuring trust and engagement of an interaction with a mobile service robot in a museum scenario. Measures were made in three different settings, either the real world, in VR or in a game-like SB and then compared with an ANOVA. The results indicate, that neither trust nor engagement differ dependent on the experimental setting. The results imply that both VR and SB are eligible ways to explore the interaction with a mobile service robot, if some peculiarities of each medium are taken into account.

## 1 Introduction

Robots are becoming more proficient in performing numerous assistive tasks like transportation and manipulation, and their social capabilities, e.g., in communication and entertainment, increase as well. With that extending range of abilities also comes a broader field of possible applications. Robots are not only used in professional environments with highly skilled and trained personnel but also in private and public spaces, populated with user groups of all kinds. This diversification of both, tasks and domains, opens up a vast, rich and complex design space for developers and researchers to explore (Schweidler et al., 2020).

Studies exploring that design space have been, and continue to be, conducted. The details of those studies vary, depending on whether they are psychological experiments, user tests, or field research—but in any case, variation of the stimulus (that is, the robot) is key to the method. This need for variation poses two challenges specific to the field of human-robot interaction (HRI): First, building and customizing robots is (still) complicated. Once an initial prototype has been built, fundamental design changes quickly become costly and difficult to implement. Second, letting humans interact with robot prototypes can be dangerous when modifications to the robot’s soft- or hardware are not rigorously tested—which again is costly and time-consuming.

To tackle these challenges a common workaround is to show subjects images or videos of robots, followed by questionnaires surveying their perception of the robot. This is problematic, since a robot presented in an image or video can be evaluated differently and might elicit a different behavior than a real robot (Li, 2015). Although researchers are aware of that loss of external validity (Bartneck, 2003; Wainer et al., 2007; Kiesler et al., 2008; Hoffmann et al., 2018), SB representations like pictures, videos or game-like scenarios are prevailing as test objects.

A promising way to solve the problems associated with either method, i.e., the use of SB representations or real robots, respectively, may lie in the use of virtual reality environments (VR). VR compared to experiments with physical comes with a relative ease of implementation and low physical risks to subjects. And although even high-fidelity VR environments are not perfect reproductions of the real world (Groom et al., 2002; Li et al., 2019), VR stands out for its immersiveness. In a VR system with a head-mounted display, the subject is presented with the images on a screen of the VR headset that is close to the eye (Bente et al., 2002), allowing a three-dimensional all-around view of the environment. As sensors on the VR headset detect the position and orientation of the head, the environment is always presented behaviorally adapted. The subject perceives rich and accurate information about their environment, especially when he or she is able to navigate the virtual environment through bodily motion. As the bodily motion becomes congruent with the (expected) change of the visual field in the simulated environment, the impression to be physically situated in this environment is reinforced (Sherman and Craig, 2018). The use of special controllers representing virtual hands within the VR environment may further increase the three-dimensional impression, as well as the feeling of being physically present within the VR environment (Schuemie et al., 2001). The virtual hands can also be used to actively change the environment (Loomis et al., 1999)—e.g., to interact directly with the VR robot. Additionally, sounds played through headphones on the VR headset enhance the three-dimensional impression (Loomis et al., 1999) and reduce distraction by external acoustic stimuli (Lum et al., 2018). The knowledge of being exposed to illusory stimuli recedes into the background, the subject has the impression of being physically situated in the virtual space, of being in the middle of it instead of just being close (Witmer et al., 2005; Sherman and Craig, 2018). There is evidence that virtual representations do not even require high naturalness to elicit valid effects (Schaefer et al., 2015). The viewer may well be aware to be confronted with an artificial environment and yet show the same behavior as in the unmediated real world (Dotsch and Wigboldus, 2008; Rinck et al., 2010). Consequently, VR environments provide the experimenter with the opportunity to observe actual, real-world-like behavior, not just to ask them about behavioral intentions as with screen-based (SB) representations. At the same time the conditions in a VR environment, as with a SB representation, can be controlled strictly to investigate the effects of the independent variables and to ensure high internal validity.

All the above intuitively makes VR a suitable way to conduct experiments in HRI and some work has been done pursuing this intuition: Studies comparing VR and SB are suggestive of a general validity and usefulness of VR as a research method (Wainer et al., 2007; Kiesler et al., 2008; Liu et al., 2017; Whitney et al., 2020). Comparing feelings towards VR vs. real robots specifically, Inoue et al. (2005) found no significant differences, while Kamide et al. (2014) found that, although subjects did not show a different behavior, they reported different feelings towards the robot in VR compared to the one in the real world (RW). Likewise, a recent study by Wijnen et al. (2020) found differences in the feelings and behavior towards a robot in VR vs. RW. However, this study did not compare the VR directly to the RW condition, instead replicating a previous RW study by Kahn et al. (2015) in VR. The authors critically note that their VR condition probably did not replicate the original study by Kahn et al. (2015) closely enough and as such perhaps caused the differences observed.

To our knowledge, however, no work has been done comparing all three different conditions, i.e., RW, VR, and SB, in one coherent paradigm, using the same robot and scenario. With the goal to provide methodological decision support for HRI researchers, we designed and conducted an experiment examining the effect of the research method on two common HRI measures: engagement and trust.

### 1.1 Engagement

Engagement is one of the central constructs of HRI (Steinfeld et al., 2006), evaluating whether a robot attracts attention and arouses a persistent interest in the interaction. This interest is especially important when the robot’s task is to convey information (Kidd and Breazeal, 2004). Studies observing that a real robot is perceived more entertaining (Wainer et al., 2007; Leite et al., 2008) and elicits more attention (Kennedy et al., 2015) than a SB-robot. According to Steinfeld et al. (2006) the VR environment offers more interaction possibilities than a SB environment, because the subject can get in contact with the robot with his virtual hands. It is hypothesized that due to these greater interaction opportunities, the subjects will be more engaged within the VR condition than in the SB condition. Consequently, it is hypothesized that:

H1. Subjects in the RW and VR condition are more engaged than subjects in the SB condition.

However, due to the novelty effect, it is assumed that the subjects initially show increased engagement and interest, which decreases over time (Smedegaard, 2019). It is therefore hypothesized that:

H2. In all three conditions engagement will decrease over time.

### 1.2 Trust

Trust is another prominent construct in HRI research (Steinfeld et al., 2006) and a central precondition for a positive interaction between human and robot to emerge (Sanders et al., 2011; van Pinxteren et al., 2019). Studies have shown that physical proximity between human and robot is an important trust building factor (Bainbridge et al., 2008; Billings et al., 2012).

The findings of Bainbridge and colleagues (2008) suggest that humans tend to trust rather a real robot than a robot presented on a screen. If a robot behaves and performs as expected, it contributes to building trust towards the robot (Kidd and Breazeal, 2004). In contrast to a robot presented on a screen, a VR robot can interact with a human in a three-dimensional space in the same way as a real robot can. Therefore, it is hypothesized that:

H2.1. Subjects in the RW and VR condition will perceive a comparable level of trust in the robot that varies from the trust perceived by subjects in the SB condition.

Due to the interaction experience with the robot it is furthermore predicted that:

H2.2. The level of trust in the robot changes over time.

To account for the contradictory findings concerning the behavior shown vs. the feelings reported (Kamide et al., 2014), both variables, engagement and trust, will be assessed through self-report and complemented by behavioral measures.

## 2 Method and material

### 2.1 Participants

34 German-speaking subjects were recruited and received a compensation of 20 Euros after participation. Four subjects were excluded due to technical problems with data logging. Our final sample included 30 subjects (15 males, 15 females), with age ranging from 19 to 78 (M = 41.73, SD = 21.03). Subjects were randomly assigned to one of the three between-subjects conditions, balancing for age and gender.

### 2.2 Scenario

The subjects experienced a tour through an art gallery, guided by a robot. When the robot and the subject arrived at a painting, the robot started telling some facts about the artwork. When the robot was done, the subject could choose to either hear more information about the current painting (the robot had been equipped with three chunks of information for each artwork) or to move on to the next painting. To avoid systematic influences of order, five different routes through the gallery were randomly assigned to the subjects.

### 2.3 Technical implementation

**FIGURE 1:**
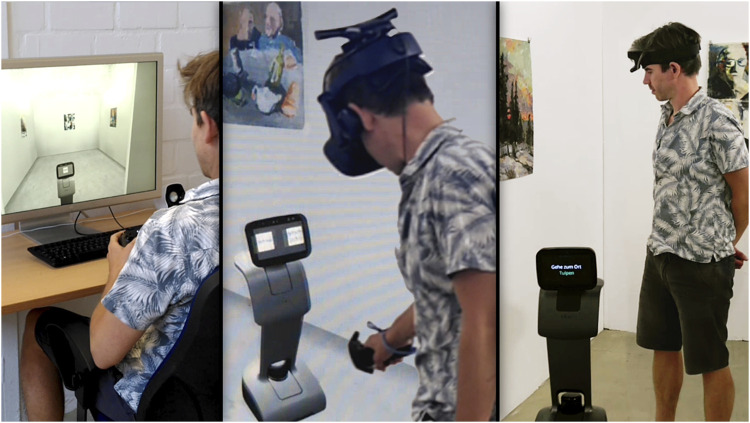
A comparative illustration of the three experimental conditions (from left to right: SB, VR and RW). The VR state image is a montage of the exterior view and an expanded view of what was shown on the VR goggles.

**FIGURE 2:**
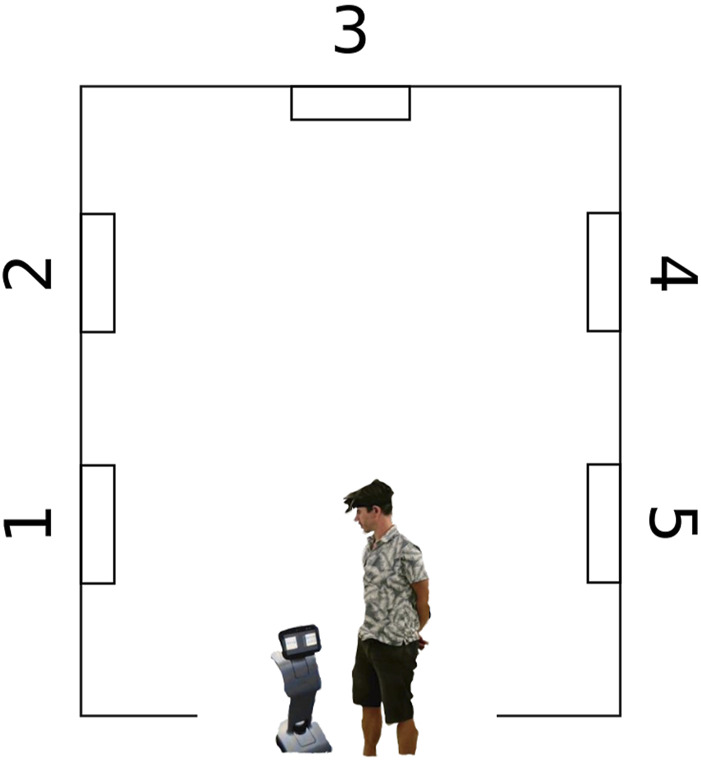
A floor plan of the gallery, indicating the 5 paintings/way points.

The study was conducted at a lab of HFC Human-Factors-Consult GmbH in Berlin. We used the robot Temi^1^, a wheeled, armless service robot equipped with a touch-screen (Figure 1). The VR and SB simulation were developed using the Unreal Engine Editor^2^ with a 3D model of the robot. We used an HTC Vive^3^ system for interaction with the VR environment. The SB simulation was presented on a 30-inch screen and stereo loudspeakers. The subjects could navigate the gallery using a gamepad^4^. The environment, the robot, and the interaction were designed as similar as possible for all three conditions (see Figure 1). All subjects experienced a typical white cube gallery measuring 6 × 5 m with five paintings (five-way points) displayed on the walls (see Figure 2). The paintings were all similar in size, style and originator.

### 2.4 Measurements

To ensure comparability of the results to other studies, established questionnaires were chosen for engagement and trust. In addition to the questionnaires, two typical behavioral measures were assessed.

The reported engagement was captured using the subscale interest/enjoyment of the Intrinsic Motivation Inventory (IMI; Deci and Ryan, 2003; German version: own translation). The inventory surveys the entertainment and interest elicited by a certain activity on a 7-point Likert scale and is often used in MRI studies (Zaga, 1995; Schuler et al., 2011). The inventory was complemented by the observed engagement, operationalized by measuring the time of each interaction, a frequently used indicator for motivation and engagement in MRI research in general (Sidner et al., 2005; Steinfeld et al., 2006; Powers et al., 2007), and especially in the context of museum robots (Bickmore et al., 2013). The interaction time was defined as the period during which the subject listened to the robot in front of a single painting (waypoint). It was assumed that the longer the interaction lasted, the more interested and engaged the subject was. The interaction time was measured for all five waypoints (within-subject factor interaction experience; see Figure 2) and compared between the three conditions (between-subject factor condition; see Figure 1).

The Trust Perception Scale-HRI questionnaire (TPS-HRI; Schaefer, 2016; German version: own translation) measures the perceived trust and was used in the short version with 14 items to capture the reported trust. Subjects could indicate how much of the time they perceived the robot as trustworthy on a 10%-stepped scale. The questionnaire is well suited to compare trust in robots between different scenarios (Schaefer, 2016). The reported data was complemented by continuously measuring the chosen distance to the robot as the distance can be interpreted as a behavioral measure for trust (Deligianis et al., 2017). The measure was averaged for the time subject and robot stood in front of the painting at each of the five-way points and will be referred to as ‘observed trust’.

### 2.5 Procedure

The subjects were randomly assigned to one of the three conditions. Prior to the experiment, they were informed about the procedure and that they could take as much time as they wanted to look at each painting. In the VR and SB condition, the experimental session was preceded by a training session, where subjects could navigate a completely empty virtual room to get familiar with the virtual environment, to adjust the VR headset to their individual needs (sharpness of the image reproduction, volume of sounds), and to get used to the game-pad. The training sessions lasted approximately 2 min.

Depending on the condition, subjects then experienced the gallery tour with the robot under one of the three conditions (RW, VR, SB). After the experiment, subjects completed the questionnaires, and received their compensation and a short debriefing.

### 2.6 Experimental design

Statistical analysis was performed using the SPSS statistical program (IBM Corp., 2013). Test power calculation was performed with the software G*Power (Faul et al., 2007). Prior to the inferential statistical analysis, the preconditions of the respective methods were examined. An exploratory data analysis did not detect any extreme outliers (defined as three times the interquartile range). The variables were tested for normal distribution using the Shapiro-Wilk test. The data were normally distributed, *p* > .05, unless otherwise reported in the results section. For data analysis, analyses of variance were mainly calculated, as they are considered to be relatively robust to violation of the normal distribution (Glass et al., 1972; Rasch and Guiard, 2004). In addition, all variables were tested for variance homogeneity using Levene’s test. When variance homogeneity was given, a not too conservative *post hoc* analysis, the Tukey test, was chosen because the focus of the study was to test the null hypothesis (comparison of the RW and VR conditions). In the case of violation of variance homogeneity, a Welch ANOVA, and for *post hoc* analyses, the Games-Howell test was applied. When sphericity was violated in repeated-measures analyses, a Greenhouse-Geisser correction was applied to the degrees of freedom.

The scores of IMI and TPS-HRI were submitted to a one-way ANOVA, to analyze the reported engagement and trust. At each of the five waypoints the subject´s behavior (interaction time and distance to the robot) was measured and later compared between the conditions. In both cases, a 3 × 5 mixed ANOVA with the between-subject factor condition (RW, VR, SB) and the within-subject factor waypoint (1, 2, 3, 4, 5) was used. In addition, the initial following distance to the robot when the subjects were guided to the first waypoint was analyzed using a one-way ANOVA.

By default, a significance level of *α* = 0.05 was assumed. Only for the *post hoc* comparison of the RW vs. VR condition a significance level of *α* = 0.20 was used, as a null hypothesis was tested.

## 3 Results

### 3.1 Control variables

To control for systematic differences between the three conditions, control variables were assessed and compared between conditions using one-way ANOVAs. No differences were found in negative attitudes towards robots (NARS), affinity for technology interaction (ATI), immersive tendency (ITQ) and interest in art. All control variables were analyzed for correlation with the perceived and demonstrated engagement and trust. No significant correlations were found.

In order to get a qualitative overview of the experiment, we observed each subject unobtrusively but not secretly. We could see that some subjects in the SB condition navigated the gallery in a random manner. After the experiment one of these subjects stated: “I still felt unsure in the beginning how to handle the gamepad”. Therefore, we had a closer explorative look at the data and our observation was confirmed: During the walk to the first waypoint (initial following distance), the SB condition with the gamepad showed a greater variance in the distance values (SB: *M* = 1.98, *SD* = 0.63) than in the other two conditions (RW: *M* = 0.74, *SD* = 0.23; VR: *M* = 0.98, *SD* = 0.20). The control variable previous experience in game-pad use did not show significant correlation with the initial following distance. However, the six subjects who had never used a game-pad before showed a higher variance on that score (*M* = 2.24, *SD* = 0.70) than the four subjects who had used a gamepad at least once (*M* = 1.58, *SD* = 0.15).

### 3.2 Engagement

The descriptive statistics of the IMI score suggest that subjects in the RW and VR condition felt more engaged compared to the subjects in the SB condition (RW: *M* = 5.20, *SD* = 1.01; VR: *M* = 5.21, *SD* = 0.91, SB: *M* = 4.56, *SD* = 1.10), yet the ANOVA results did not support that impression [*F* (2, 27) = 1.38, *p* = .268, η^2^_p_ = .09, power = 27.1 %]. For the behavioral engagement, the descriptive data indicated that on average, for the five-way points, the subjects in the RW condition listened to the robot the longest, followed by those in the VR and SB condition. However, there was no evidence for a statistical significant effect [*F* (2, 27) = 2.51, *p* = .100, η^2^ *p* = .16, power = 67.3 %], so the hypothesis that subjects in the RW and VR condition are more engaged than subjects in the SB condition (H1.1) is rejected. No evidence was found for a main effect of way point, *F* (4, 108) = .59, *p* = .673, η^2^_p_ = .02, power = 59.7%. The three conditions showed no changes in their interaction time across the five-way points, which leads us to reject the hypothesis that in all three conditions engagement will decrease over time (H1.2).

### 3.3 Trust

The descriptive values of the TPS-HRI show the lowest ratings in the VR condition (*M* = 78.79, *SD* = 8.45), followed by the SB condition (*M* = 81.71, *SD* = 7.14) and the RW condition (*M* = 83.86, *SD* = 12.50). Again, the difference between the three conditions was not statistically significant (*F* (2, 27) = 0.70, *p* = .506, η^2^_p_ = .05, power = 15.5 %). Concerning the observed trust, the first measure analyzed was the initial value, i.e., the average distance between human and robot when the subjects were guided to the first waypoint. Shapiro-Wilk tests revealed that the normality assumption was violated for condition SB, indicating a higher probability of type II error. However, a one-way factorial ANOVA showed a statistically significant difference between the three conditions, Welch-test *F* (2, 16.45) = 17.01, *p* < .001, η^2^_p_ = .66, power = 99%. For *post hoc* comparisons, see Table 1.

**TABLE 1:** Games-Howell *post hoc* test - Initial following distance and distances per way point from subject to robot (in meters). The mean difference is significant at the .05 level for the comparison of the RW vs. SB and VR vs. SB condition. For the comparison of the RW vs. VR condition a significance level of *α* = 0.20 is used.

						95% confidence interval for difference	
	Waypoint		*M*	*SE*	*p*	Lower bound	Upper bound
Initial following Distance		RW - VR	-,24	,10	,057	-,48	,01
		RW - SB	−1.24	,21	,000	−1.80	-,67
		VR - SB	-,10	,21	,002	−1.56	-,44
Distances per Waypoint	1	RW - VR	-,11	,07	,217	-,28	,05
		RW - SB	-,87	,13	,000	−1.21	-,52
		VR - SB	-,75	,13	,000	−1.09	-,41
	2	RW - VR	,08	,07	,580	-,12	,27
		RW - SB	-,50	,19	,054	−1.01	,01
		VR - SB	-,57	,19	,028	−1.08	−06
	3	RW - VR	-,06	,06	,585	-,21	,09
		RW - SB	-,30	,17	,228	-,77	,17
		VR - SB	-,24	,17	,367	-,71	,23
	4	RW - VR	-,05	,06	,766	-,22	,12
		RW - SB	-,28	,09	,021	-,52	-,04
		VR - SB	-,24	,08	,032	-,45	-,02
	5	RW - VR	-,01	,09	,992	-,24	,22
		RW - SB	-,12	,15	,716	-,52	,28
		VR - SB	-,11	,15	,753	-,51	,29

**FIGURE 3:**
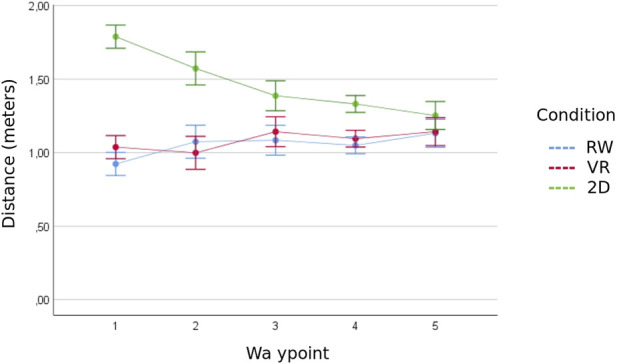
Distance between subject and robot for each condition across the five interactions. Mean values with +/- 1 standard deviation.

The following section presents the distances per waypoint to analyze the development of trust over time. The Shapiro-Wilk test were violated for waypoints 2 (of SB condition), 4 (of RW condition), and 5 (of VR condition). Levene’s test was violated for four of the five waypoints (*p* < .05). Homogeneity of the covariance matrices was not given according to the Box test (*p* < .001). Mixed ANOVA showed a statistically significant interaction between the factors condition and waypoint, Greenhouse-Geisser *F* (4.89, 65.96) = 4.71, *p* = .001, η^2^_p_ = .26, power = 91.5%. Figure 3 illustrates the means and standard deviations of the three conditions.

Evidence was found for a significant main effect of condition, *F* (2, 27) = 14.06, *p* < .001, η^2^_p_ = .51, power = 99.8%. The results of the Games-Howell *post hoc* test are shown in Table 1. As predicted, the VR condition did not differ from the RW condition at all five-way points. While the SB condition differed from the other two conditions at the first way points, no difference between all three conditions was observable at the last way point. No clear evidence could be provided for H2.1, as there are no more differences between the three conditions at the end of the recorded time span. H2.1, stating that the SB condition will differ in the level of trust, and that only RW and VR will be the same, is therefore rejected.

No evidence was found for a main effect of way point, Greenhouse-Geisser *F*(2.44, 65.96) = .672, *p* = .543, η^2^_p_ = .02, power = 52%. The three conditions showed no changes in their distances to the robot across the five-way points. Games-Howell *post hoc* tests showed a significant difference only in the SB condition between way point 1 and 4 (0.46, *p* = .021). Thus, the hypothesis that the level of trust in the robot changes over time (H2.2) is rejected.

## 4 Discussion

We designed and conducted an experiment to compare the effect of three experimentation methods, i.e., real world, virtual reality and screen based, on trust and engagement in an interaction with a mobile service robot. The main hypothesis was that real world and virtual reality will produce similar results, whereas the screen-based paradigm will yield lower ratings in trust and engagement. While the first part of that hypothesis is supported by the findings, the second part is not. There was no evidence for an effect of the experimentation method used on the perceived or demonstrated engagement, or perceived trust. Neither did the demonstrated trust, measured by the distance participants took to the robot, differ effectively between any of the conditions. It is noteworthy, though, that the initial distance participants took to the robot was in fact different for the SB compared to other conditions in the beginning of the experiment, but converged over time. These results are difficult to interpret, as non-significant differences can always be due to an insufficient test-power. However, the fact that the differences between VR and RW are not large enough to become significant at an alpha threshold of .20, can be seen as a clear indicator that the results obtained under either condition are very similar.

In the questionnaire data, the descriptive patterns of the PQ and IMI indicate that the use of a VR robot instead of a SB robot may lead to more valid results. However, at least with the present test power, the difference was not statistically significant. In particular for the self-report measures, a new study with more subjects would be meaningful, to further its significance. The TPS-HRI is not considered due to its questionable internal reliability in this study (Cronbach’s *α* = .64).

In the SB condition, subjects could not use their own bodies to move through the gallery, as in the RW and VR condition. Instead, they could only navigate their point of view through the gallery using a gamepad. This way of “locomotion” is less intuitive. During the walk to the first waypoint (initial following distance), the SB condition showed a greater variance in the distance values than in the other two conditions. To examine this descriptive finding, the correlation of the control variable *previous experience in gamepad use* with *initial following distance* was tested. The correlation test was not significant. Since the SB condition consisted of only 10 subjects, results should be interpreted with caution. However, descriptive statistics indicate that especially the subjects who had never used a gamepad before (*N* = 6) showed a higher variance in their distance values than subjects who had used a gamepad before (*M* = 2.24, *SD* = 0.70 and *M* = 1.58, *SD* = 0.15).

Another explanation why subjects in the SB condition initially maintained a greater distance might be due to the limited field of view of the SB representation. Two subjects explained that they had maintained a greater distance in order to be able to see both the painting and the robot on the screen. The subjects did not have the feeling that the robot was next to them when they were standing close to the painting, as in the VR and RW conditions—simply because the robot was outside their field of view. These statements of the subjects are a further explanation for the greater distance in the SB condition. The repetitive study design may explain why subjects navigated closer with the gamepad with each painting: They were now aware that the robot would always position himself to their right.

In all three conditions, the paintings were equally positioned and accessible from all sides. Nevertheless, there may have been a default position where the paintings in the gallery were best seen which influenced the position of the subject regardless of the presence of the robot. In a further study it would be interesting to compare the distances with and without the robot. It could help to identify more precisely the peculiarities of the HRI, independently of the systematic comparison of the three conditions that our study addressed. A qualitative difference between the conditions was the use of the robot display. In the RW and VR condition, the subjects had to approach the robot to handle the display with their real or virtual hands. This was not required in the SB condition, as the interaction was controlled by the buttons on the gamepad. This difference is not considered a methodological shortcoming of the study design. Rather, it reflects the inherent characteristics of the technologies the study was exploring. The more informative is the finding that, despite the different ways of handling the robot’s display, the subjects in the VR and SB condition chose a comparable distance to the robot as in the RW condition after some time.

Differences like these possibly influence the distance between humans and robots regardless of trust. Our study does not claim to explore the reasons why trust in robots develops. Rather, we would like to systematically compare whether distance behavior, as a common operationalization in HRI for trust, is similar across the three conditions. A VR environment seems more useful than a SB representation to obtain valid results on distance behavior already at the beginning of the experiment. In SB, subjects need more time to get used to locomotion and gaze positions. This finding is generally applicable to the survey of distances, as distance is also used as an operationalization of other constructs, such as respect (Bainbridge et al., 2008; Takayama and Pantofaru, 2009).

The study is characterized by a careful implementation of the requirements for a direct comparison between the experimental conditions. In addition to an identical visual representation of the gallery and the robot, comparable auditory stimuli were presented (e.g., the engine sound of the robot).

The common finding, that SB robots were rated differently from real robots (Wainer et al., 2007; Kiesler et al., 2008; Hoffmann et al., 2018) cannot be replicated in the present study. This may be due to the fact that in most of these studies, the SB robots are presented as photographs or video recordings (e.g., Wainer et al., 2007). These robots, unlike the SB robot that we presented in our study in a computer game, do not offer direct interaction with one another. Our results indicate that interactive SB representations, presented in a similar way to computer games, may be used equivalently to VR and RW environments to measure behavioral responses.

The findings of the distance behavior partly explain the mixed findings from previous studies. While Kamide et al. (2014) showed that no significantly different distance is taken to a VR and real robot, the study of Li et al. (2019) indicated the opposite finding. Our study showed that the differences between the VR and RW are only present at the beginning, on the way to waypoint 1. Measuring the distance only once would have supported the findings by Li et al. (2019). Only the repeated measurement revealed that the distances in all three groups converge in the long term. Our results suggest that with increasing experience with the technology, differences between groups diminish.

Although the initial implementation of the virtual world that was used for the VR and the SB testing was laborious, subsequent adaptions and adjustments to the environment, the robot, and the interaction could be done quickly and without much effort. This variability and the findings of our study make interactive simulations a very interesting option for HRI experiments, especially compared to non-interactive screen-based designs.

Since only five points in time were sampled, the explanatory power of the study is limited. It cannot be proven that the different distances at the beginning were training/familiarization effects in the SB (and VR) condition. A prolonged test or higher resolution time series would be helpful to explore the unfolding of the distance values over time. For further studies, it would be advisable to schedule longer training sessions to minimize initial differences, especially when a game pad is being used.

Due to the inherent properties of the technical devices, the distances are not directly comparable between the conditions. As the subjects in the VR and SB conditions interacted with a virtual robot, the robot was able to pass through the subjects’ body and the subject was able to pass through the robots’ body. Even though neither was observed, this knowledge may have influenced the distancing behavior.

An obvious limitation of our study is the small sample size. This was mainly caused by COVID-19 restrictions taking effect right with the beginning of our study. We tried to mitigate the effect by at least balancing the numbers of subjects for each condition. Nonetheless, this hampers the interpretation of results and should be addressed in a subsequent study. We focused on the analysis of the behavioral measures. A subsequent study with higher test power would be especially interesting for the self-report measures to find out more about the perception of the three conditions.

Given all the limitations, it is difficult to draw any general conclusions. The results can, however, be seen as an indicator, that both, virtual reality environments as well as screen-based game-like scenarios are appropriate ways to explore a close interaction between human and a mobile service robot. The results further show, that when screen-based HRI experiments are to be conducted, participants must be given sufficient time to accommodate to the interaction technology, otherwise it might mask the interaction between human and robot. Otherwise, screen-based interaction paradigms appear to be more eligible if only reported instead of behavioral measures are to be explored and should be implemented carefully and as interactively as possible. VR has proven to being able to produce results quite similar to an experiment in the real world for both behavioral and reported measures. However, to make more general claims, additional studies with higher numbers of participants and with a greater variety of HRI scenarios must follow.

## Data Availability

The raw data supporting the conclusion of this article will be made available by the authors, without undue reservation.

## Footnotes

1
https://www.robotemi.com/.

2
https://www.unrealengine.com/en-US/.

3
https://www.vive.com/de/.

4
https://www.xbox.com/de-DE/accessories/controllers/xbox-wireless-con- troller.

## References

[B1] Bainbridge W. A. Hart J. Kim E. S. Scassellati B. (2008). “The effect of presence on human-robot interaction,” in RO-MAN 2008-the 17th IEEE international symposium on robot and human interactive communication (IEEE), 701–706.

[B2] Bartneck C. (2003). “Interacting with an embodied emotional character,” in Proceedings of the 2003 international conference on Designing pleasurable products and interfaces, 55–60.

[B3] Bente G. Krämer N. C. Petersen A. (2002). Virtuelle realitäten. Hogrefe Verlag.

[B4] Bickmore T. W. Vardoulakis L. M. P. Schulman D. (2013). Tinker: A relational agent museum guide. Aut. agents multi-agent Syst. 27 (2), 254–276. 10.1007/s10458-012-9216-7

[B5] Billings D. R. Schaefer K. E. Chen J. Y. Hancock P. A. (2012). “Human-robot interaction: Developing trust in robots,” in Proceedings of the seventh annual ACM/IEEE international conference on Human-Robot Interaction, 109–110.

[B6] Deci E. L. Ryan R. M. (2003). Intrinsic motivation inventory. CSDT. Retrieved 09.09.2022, from. Available at: https://selfdeterminationtheory.org/intrinsic-motivation-inventory/ .

[B7] Deligianis C. Stanton C. J. McGarty C. Stevens C. J. (2017). The impact of intergroup bias on trust and approach behaviour towards a humanoid robot. J. human-robot Interact. 6 (3), 4–20. 10.5898/jhri.6.3.deligianis

[B8] Dotsch R. Wigboldus D. H. J. (2008). Virtual prejudice. J. Exp. Soc. Psychol. 44 (4), 1194–1198. 10.1016/j.jesp.2008.03.003

[B9] Faul F. Erdfelder E. Lang A. G. Buchner A. G. (2007). G power 3: A flexible statistical power analysis program for the social, behavioral, and biomedical sciences. Behav. Res. Methods 39 (2), 175–191. 10.3758/BF03193146 17695343

[B10] Glass G. V. Peckham P. D. Sanders J. R. (1972). Consequences of failure to meet assumptions underlying the fixed effects analyses of variance and covariance. Rev. Educ. Res. 42 (3), 237–288. 10.3102/00346543042003237

[B11] Groom C. J. Sherman J. W. Conrey F. R. (2002). What immersive virtual environment technology can offer to social cognition. Psychol. Inq. 13 (2), 125–128.

[B12] Hoffmann L. Bock N. vd Pütten A. M. R. (2018). “The peculiarities of robot embodiment (EmCorp-Scale): Development, validation and initial test of the embodiment and corporeality of artificial agents scale,” in 2018 13th ACM/IEEE international conference on human-robot interaction (HRI) (IEEE), 370–378.

[B13] Inoue K. Nonaka S. Ujiie Y. Takubo T. Arai T. (2005). “Comparison of human psychology for real and virtual mobile manipulators,” in ROMAN 2005. IEEE international workshop on robot and human interactive communication, 2005 (IEEE), 73–78.

[B14] Kahn P. H. Jr Kanda T. Ishiguro H. Gill B. T. Shen S. Gary H. E. (2015). “Will people keep the secret of a humanoid robot? Psychological intimacy in HRI,” in Proceedings of the tenth annual ACM/IEEE international conference on human-robot interaction, 173–180.

[B15] Kamide H. Mae Y. Takubo T. Ohara K. Arai T. (2014). Direct comparison of psychological evaluation between virtual and real humanoids: Personal space and subjective impressions. Int. J. Human-Computer Stud. 72 (5), 451–459. 10.1016/j.ijhcs.2014.01.004

[B16] Kennedy J. Baxter P. Belpaeme T. (2015). Comparing robot embodiments in a guided discovery learning interaction with children. Int. J. Soc. Robotics 7 (2), 293–308. 10.1007/s12369-014-0277-4

[B17] Kidd C. Breazeal C. (2004). “Effect of a robot on user perceptions,” in 2004 IEEE/RSJ International conference on intelligent robots and systems, 4. 10.1109/IROS.2004.1389967

[B18] Kiesler S. Powers A. Fussell S. R. Torrey C. (2008). Anthropomorphic interactions with a robot and robot–like agent. Soc. Cogn. 26 (2), 169–181. 10.1521/soco.2008.26.2.169

[B19] Leite I. Pereira A. Martinho C. Paiva A. (2008). “Are emotional robots more fun to play with?,” in RO-MAN 2008-the 17th IEEE international symposium on robot and human interactive communication (IEEE), 77–82.

[B20] Li J. (2015). The benefit of being physically present: A survey of experimental works comparing co-present robots, telepresent robots and virtual agents. Int. J. Human-Computer Stud. 77, 23–37. 10.1016/j.ijhcs.2015.01.001

[B21] Li R. van Almkerk M. van Waveren S. Carter E. Leite I. (2019). “Comparing human-robot proxe-mics between virtual reality and the real world,” in 2019 14th ACM/IEEE international con ference on human-robot interaction (HRI) (IEEE), 431–439.

[B22] Liu O. Rakita D. Mutlu B. Gleicher M. (2017). “Understanding human-robot interaction in virtualreality,” in 2017 26th IEEE international symposium on robot and human interactive communication (RO-MAN) (IEEE), 751–757.

[B23] Loomis J. M. Blascovich J. J. Beall A. C. (1999). Immersive virtual environment technology as a basic research tool in psychology. Behav. Res. methods, Instrum. Comput. 31 (4), 557–564. 10.3758/bf03200735 10633974

[B24] Lum H. C. Greatbatch R. Waldfogle G. Benedict J. (2018). How immersion, presence, emotion, & workload differ in virtual reality and traditional game mediums. Proc. Hum. Factors Ergonomics Soc. Annu. Meet. 62 (1), 1474–1478. 10.1177/1541931218621334

[B25] Powers A. Kiesler S. Fussell S. Torrey C. (2007). “Comparing a computer agent with a humanoid robot,” in Proceedings of the ACM/IEEE international conference on Human-robot interaction, 145–152.

[B26] Rinck M. Rörtgen T. Lange W.-G. Dotsch R. Wigboldus D. H. J. Becker E. S. (2010). Social anxiety predicts avoidance behaviour in virtual encounters. Cognition Emot. 24 (7), 1269–1276. 10.1080/02699930903309268

[B27] Sanders T. Oleson K. E. Billings D. R. Chen J. Y. C. Hancock P. A. (2011). A model of human-robot trust: Theoretical model development. Proc. Hum. Factors Ergonomics Soc. Annu. Meet. 55 (1), 1432–1436. 10.1177/1071181311551298

[B28] Schaefer K. E. (2016). “Measuring trust in human robot interactions: Development of the “trust perception scale-HRI”,” in Robust intelligence and trust in autonomous systems (Boston, MA: Springer), 191–218.

[B29] Schaefer K. E. Sanders T. Kessler T. T. Dunfee M. Wild T. Hancock P. A. (2015). “Fidelity & validity in robotic simulation,” in 2015 IEEE international inter-disciplinary conference on cognitive methods in situation awareness and decision support (CogSIMA) (Orlando, FL: IEEE), 113–117. 9-12 March 2015. 10.1109/COGSIMA.2015.7108184

[B30] Schuemie M. J. van der Straaten P. Krijn M. van der Mast C. A. (2001). Research on presence in virtual reality: A survey. CyberPsychology Behav. 4 (2), 183–201. 10.1089/109493101300117884 11710246

[B31] Schuler T. Brütsch K. Müller R. van Hedel H. J. A. Meyer-Heim A. (2011). Virtual realities as motivational tools for robotic assisted gait training in children: A surface electromyography study. NeuroRehabilitation 28 (4), 401–411. 10.3233/NRE-2011-0670 21725175

[B32] Schweidler P. Tausch A. Oehme A. Jürgensohn T. (2020). MRI-Szenarien einfach klassifizieren mit dddder Kontext-Person-Roboter-Heuristik „KOPROH.

[B33] Sherman W. R. Craig A. B. (2018). Understanding virtual reality: Interface, application, and design. Elsevier Science.

[B34] Sidner C. L. Lee C. Kidd C. D. Lesh N. Rich C. (2005). Explorations in engagement for humans and robots. Artif. Intell. 166 (1-2), 140–164. 10.1016/j.artint.2005.03.005

[B35] Smedegaard C. V. (2019). “Reframing the role of novelty within social HRI: From noise to information,” in 2019 14th ACM/IEEE international conference on human-robot interaction (HRI) (IEEE), 411–420.

[B36] Steinfeld A. Fong T. Kaber D. Lewis M. Scholtz J. Schultz A. (2006). “Common metrics for human-robot interaction,” in Proceedings of the 1st ACM SIGCHI/SIGART conference on Human-robot interaction, 33–40.

[B37] Takayama L. Pantofaru C. (2009). “Influences on proxemic behaviors in human-robot interaction,” in 2009 IEEE/RSJ international conference on intelligent robots and systems (IEEE), 5495–5502.

[B38] van Pinxteren M. E. Wetzels R. W. H. Rüger J. Pluymaekers M. Wetzels M. (2019). Trust in humanoid robots: Implications for services marketing. J. Serv. Mark. 33 (4), 507–518. 10.1108/JSM-01-2018-0045

[B39] Wainer J. Feil-Seifer D. J. Shell D. A. Mataric M. J. (2007). “Embodiment and human-robot interaction: A task-based perspective,” in RO-MAN 2007-the 16th IEEE international symposium on robot and human interactive communication (IEEE), 872–877.

[B40] Whitney D. Rosen E. Phillips E. Konidaris G. Tellex S. (2020). “Comparing robot grasping tele-operation across desktop and virtual reality with ROS reality,” in Robotics research (Cham: Springer), 335–350.

[B41] Wijnen L. Lemaignan S. Bremner P. (2020). “Towards using Virtual Reality for replicating HRI studies,” in Companion of the 2020 ACM/IEEE international conference on human-robot interaction, 514–516.

[B42] Witmer B. G. Jerome C. J. Singer M. J. (2005). The factor structure of the presence questionnaire. Presence Teleoperators Virtual Environ. 14 (3), 298–312. 10.1162/105474605323384654

[B43] Zaga J. (1995). Presence, Telepresence and immersion: The cognitive factors of embodiment and interaction in virtual environments. UK Queen Mary and Westfield University of London.

